# Copeptin as a marker of an altered CRH axis in pituitary disease

**DOI:** 10.1007/s12020-017-1366-6

**Published:** 2017-08-09

**Authors:** Krzysztof C. Lewandowski, Andrzej Lewiński, Elżbieta Skowrońska-Jóźwiak, Katarzyna Malicka, Wojciech Horzelski, Georg Brabant

**Affiliations:** 1 Department of Endocrinology and Metabolic Diseases, Medical University of Lodz, Lodz, Poland; 2 Polish Mother’s Memorial Hospital—Research Institute, Lodz, Poland; 3 Faculty of Mathematics and Computer Science, University of Lodz, Lodz, Poland; 4 Experimental and Clinical Endocrinology Med Clinic I, University of Luebeck, Ratzeburger Allee 160, 23538 Lübeck, Germany

**Keywords:** Copeptin, CRH test, Pituitary, Diabetes insipidus

## Abstract

**Background:**

Copeptin (pre-proAVP) secreted in equimolar amounts with vasopressin closely reflects vasopressin release. Copeptin has been shown to subtly mirror stress potentially mediated via corticotrophin-releasing hormone. To further test a potential direct interaction of corticotrophin-releasing hormone with copeptin release, which could augment vasopressin effects on pituitary function, we investigated copeptin response to corticotrophin-releasing hormone.

**Patients and methods:**

Cortisol, adrenocorticotropin and copeptin were measured in 18 healthy controls and 29 subjects with a history of pituitary disease during standard corticotrophin-releasing hormone test.

**Results:**

Patients with previous pituitary disease were subdivided in a group passing the test (P1, *n* = 20) and failing (P2, *n* = 9). The overall copeptin response was higher in controls than in subjects with pituitary disease (area under the curve, *p* = 0.04 for P1 + P2) with a maximum increase in controls from 3.84 ± 2.86 to 12.65 ± 24.87 pmol/L at 30 min, *p* < 0.05. In contrast, both groups of pituitary patients lacked a significant copeptin response to corticotrophin-releasing hormone, and even in P1, where adrenocorticotropin concentrations increased fourfold (mean, 21.48 vs. 91.53 pg/mL, *p* < 0.01), copeptin did not respond (e.g., 4.35 ± 5.81 vs. 5.36 ± 6.79 pmol/L, at 30 min, *p* = ns).

**Conclusions:**

Corticotrophin-releasing hormone is able to stimulate copeptin release in healthy controls suggesting a direct interaction of corticotrophin-releasing hormone and vasopressin/vasopressin. Interestingly, this relation is altered already in the group of pituitary patients who pass the standard corticotrophin-releasing hormone test indicating (1) the corticotrophin-releasing hormone–adrenocorticotropin–cortisol response is largely independent from the vasopressin system, but (2) the corticotrophin-releasing hormone–vasopressin interaction reflected by copeptin may be much more sensitive to reveal subtle alterations in the regulation of pituitary function.

## Introduction

Assessment of anterior pituitary function during dynamic tests (insulin tolerance test (ITT) or glucagon stimulation test (GST)) involves estimation of adrenocorticotropin (ACTH)–cortisol secretion as well as growth hormone [1]. Contribution of vasopressin (AVP) to cortisol release was postulated for many years [2]. In particular, AVP, can both independently stimulate ACTH release as well as potentiate the effects of corticotrophin-releasing hormone (CRH) [3, 4]. AVP is produced by neurons of the hypothalamic paraventricular and supraoptic nuclei that are organised into two major systems: magnocellular and parvocellular [4]. While AVP of magnocellular origin is primarily responsible for water conservation in the kidney, and regulation of its secretion depends upon osmotic stimulation, parvocellular AVP expression and secretion is independent of the osmotic status and increases during stress [5]. Stress-related actions of AVP are thought to be mediated primarily by the, G-protein-coupled, pituitary V1b receptor [6]. Acute stress, including hypoglycaemia, leads to rapid release of CRH and AVP into the pituitary portal circulation [7], while studies in sheep and horse have shown rapid and equal elevations of CRH and AVP in the pituitary portal circulation following stress [8, 9].

Despite these data, direct interactions between AVP and CRH on the hypothalamic/pituitary levels are not fully elucidated. This was, at least partially, related to problems with AVP measurements in human subjects. In particular, plasma concentrations of AVP are technically difficult to determine due to the small molecular size and its avid binding to platelets [10, 11]. Recently, however, these problems have been largely overcome by the measurements of copeptin.

Copeptin (or C-terminal provasopressin), a glycosylated 39-amino-acid peptide, is a product of proteolysis of the AVP precursor, that is processed to AVP, neurophysin II and copeptin in equimolar amounts [12]. In contrast to AVP, copeptin remains stable for several days at room temperature in serum or plasma [13]. This formed the hypothesis that measurements of copeptin concentrations closely and reliably reflect AVP concentrations. A number of studies confirmed this hypothesis showing close parallelity between AVP and copeptin under different physiological and pathophysiological conditions [14, 15]. Thus, copeptin may serve as a bona fide biomarker of AVP release based on large studies and may be useful to distinguish in some circumstances among different causes of diabetes insipidus [16]. Copeptin levels were measured both during ITT [17, 18] and in patients with type 1 diabetes during hypoglycaemia [19]. In both circumstances, an increase in copeptin concentrations after hypoglycaemia was clearly demonstrated. Recently, we have also demonstrated an unequivocal increase in copeptin concentrations during a glucagon stimulation test [20], i.e., another well-recognised test of anterior pituitary function, that, like ITT, involves assessment of both cortisol and growth hormone secretion [1]. There was also a significant, though moderate, correlation between copeptin and ACTH concentrations. It was, however, not clear whether glycaemic fluctuations during GST result in release in CRH that in turn stimulates vasopressin/copeptin release, subsequently leading to ACTH secretion, or whether AVP/copeptin release during the test represents a phenomenon that is largely independent of CRH stimulation. Therefore, we designed a study, where we assessed direct effects of CRH on the release of AVP/copeptin as well as ACTH and cortisol. To the best of our knowledge, direct effects of CRH administration on serum copeptin concentrations have not been studied, so far.

## Patients and methods

The study involved 47 subjects (12 males), age 43.87 ± 17.6 (mean ± SD), BMI including 18 healthy controls (age 40.72 ± 18.9 years) and 29 subjects with a history of pituitary disease, age 45.82 ± 16.7 years. Human CRH (CRH Ferring^®^) was administered intravenously at the dose of 100 µg, while concentrations of copeptin, ACTH and cortisol were performed at −15, 0, 15, 30, 60 and 90 min. As CRH test was used to assess the integrity of ACTH–cortisol axis, and not as a test for a differential diagnosis of ACTH-dependent Cushing’s syndrome, we defined a successful CRH test result as cortisol concentration above 450 nmol/L (16.25 µg/dL) at any time during the test. We have selected an identical cortisol cutoff as used during a GST during our previous study of copeptin secretion during GST [20] and according to the study of Böttner et al. [21], where this cutoff value provided the best balance of sensitivity (88.5%) and specificity (86.8%) for the GST.

Patients with previous pituitary disease were subdivided in a group passing the test (P1, *n* = 20) and failing (P2, *n* = 9). The latter group included five patients with diabetes insipidus. The list of diagnoses included the following, for group P1: non-secreting adenoma, *n* = 8; acromegaly (after surgery), *n* = 2; Cushing’s disease after surgery, *n* = 2; head trauma, *n* = 2; histiocytosis, *n* = 1; and prolactinoma, *n* = 6 and for group P2: craniopharyngioma, *n* = 3; suspected pituitary gene mutations, *n* = 2; acromegaly (after surgery), *n* = 1; isolated ACTH deficiency, *n* = 1; and non-secreting pituitary adenoma, *n* = 1.

Patients from group P1 did not receive any hormonal medication with exception of cabergoline in cases of prolactinoma. Patients from group P2 received replacement therapy with hydrocortisone, thyroxine, sex steroids (*n* = 7) and desmopressin (*n* = 5). Morning hydrocortisone dose was omitted on the day of the test.

Measurements of cortisol and other hormones, including free T4, free T3, TSH, LH, FSH, prolactin, testosterone and oestradiol, were performed by immunoassays on Roche Diagnostics COBAS e601 platform, while ACTH was measured by immunoassays on Siemens IMMULITE 2000 XPi platform. Copeptin was measured with a sandwich immunoassay, as described before [13, 22]. This assay has a lower detection limit of 0.4 pmol/L; functional assay sensitivity at <20% interassay CV, <1 pmol/L. All samples were assayed as a batch analysed in one run.

The study has been approved by the Ethics Committee of the Polish Mother’s Memorial Hospital—Research Institute (Decision no. 74/2016). Informed consent was obtained from all patients participating in the study.

### Statistical analysis

Statistical analysis was performed by means of MedCalc Software 12.6.1 software. Analysis of measured covariates was performed both by serial measurements method (area under the curve calculation) and by ANOVA at distinct time points following CRH stimulation. For nonparametric data, Kruskal–Wallis test was used instead. Correlation analyses were performed using Pearson coefficient or Spearman’s rank correlation. Wilcoxon test for paired samples was used for comparison of the parameters’ values ​​for different times of measurement.

*P* values of 0.05 were considered to indicate statistical significance.

## Results

There were no significant differences in age between controls and patients (P1 + P2), *p* = 0.34. Results of serial analysis of copeptin, ACTH and cortisol secretion (area under the curve) are presented in Table 1. Serial measurement analysis revealed significantly higher overall copeptin secretion in controls vs. patients (P1 + P2), *p* = 0.039, mostly due to significantly higher levels in controls vs. P2 (*p *= 0.035), while overall copeptin secretion in controls was non-significantly higher than in P1 (*p*= 0.13). As expected, cortisol and ACTH secretion was significantly lower in P2 vs. controls and P1, however, there was no difference in ACTH or cortisol secretion between controls and P2 (*p*= 0.55 and  0.87, respectively).

**Table 1:** Comparison of area under the curve analysis (arbitrary units) of 47 patients who had CRH test (100 µg iv), including controls (*n* = 18) and patients (P1 + P2, *n* = 29) subdivided into subjects who passed CRH test (defined as cortisol concentrations above 450 nmol/L (16.25 µg/dL))—P1 (*n* = 20), and those who failed CRH test—P2 (*n* = 9)

Parameter	Group	Mean area under the curve	95% CI	SD	Median	*p* (vs. C)
Copeptin	Control	761.67	263.5 to 1259.7	968.7	540.4	–
	P1 + P2	410.78	203.6 to 617.9	512.9	259.6	0.039
	P1	469.02	174.6 to 763.3	591.8	275.7	0.139
	P2	279.76	74.05 to 485.47	246.0	179.1	0. 035
Cortisol	Control	2126.12	1910.3 to 2341.9	433.9	2020.0	–
	P1 + P2	1778.96	1364.2 to 2193.6	1048.3	1731.6	0.191
	P1	2163.70	1748.1 to 2579.2	887.8	1951.5	0.871
	P2	679.70	122.3 to 1237.1	602.7	852.5	<0.001
ACTH	Control	5155.07	3737.9 to 6572.1	2849.6	4661.4	–
	P1 + P2	4702.98	2537.2 to 6868.7	5693.6	3159.7	0.134
	P1	5483.89	2424.6 to 8543.1	6536.6	3160.8	0.553
	P2	2967.62	923.3 to 5011.9	2659.5	2188.5	0.008

**Fig. 1:**
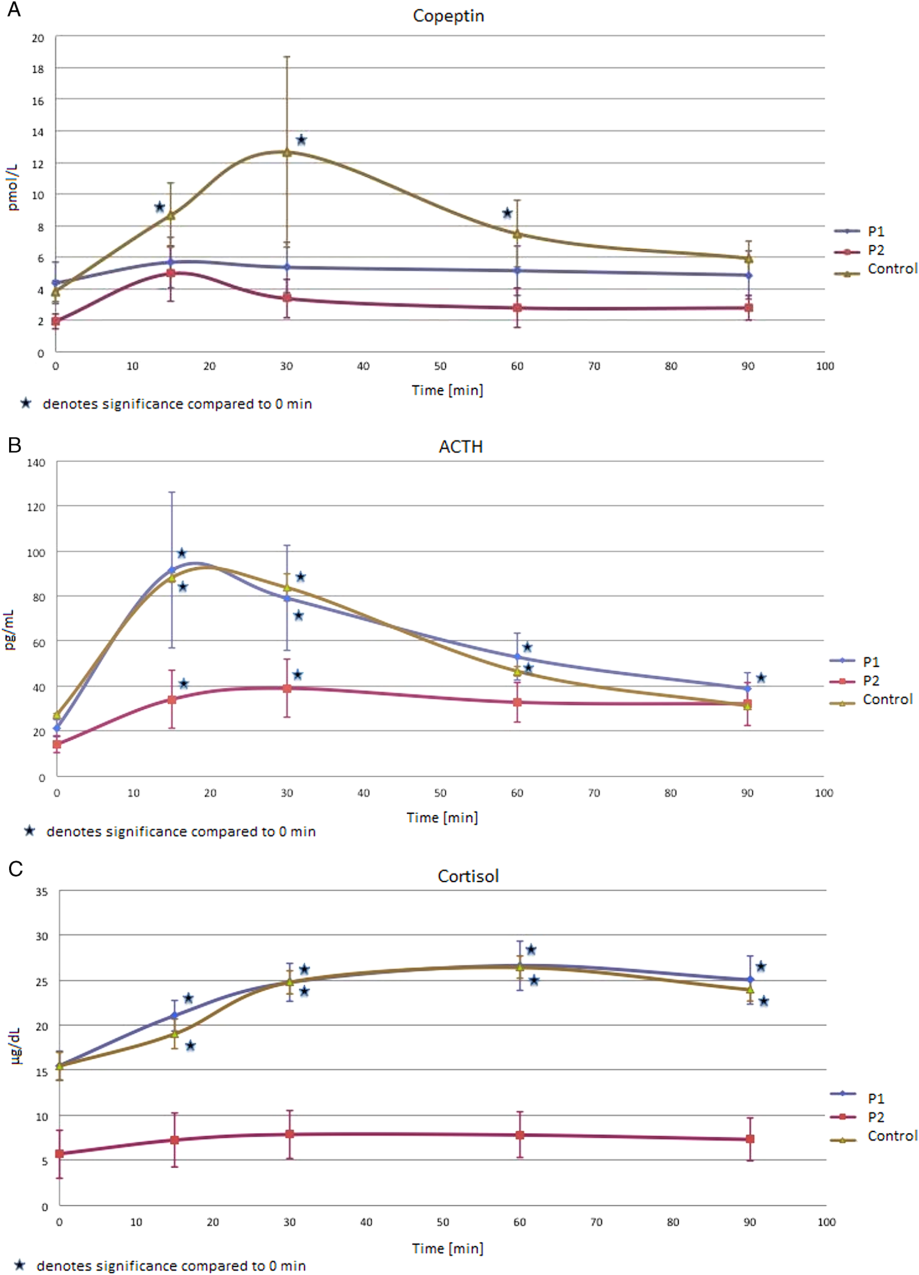
**a** Copeptin concentrations (pmol/L) during CRH test of 47 patients, including controls (*n* = 18), and patients (P1 + P2, *n* = 29), subdivided into subjects who passed CRH test (defined as cortisol concentrations above 450 nmol/L (16.25 µg/dL))—P1 (*n* = 20), and those who failed CRH test—P2 (*n* = 9). Significant increase in copeptin in comparison to time 0 min (*p* < 0.05) is marked by *. **b** ACTH concentrations (pg/mL) during CRH test of 47 patients, including controls (*n* = 18), and patients (P1 + P2, *n* = 29), subdivided into subjects who passed CRH test (defined as cortisol concentrations above 450 nmol/L (16.25 µg/dL))—P1 (*n* = 20), and those who failed CRH test—P2 (*n* = 9). Significant increase in ACTH in comparison to time 0 min (*p* < 0.05) is marked by *. An increase in ACTH concentrations in controls and P1 at 15 and 30 min of CRH was highly significant (*p* < 0.01). **c** Cortisol concentrations (µg/dL) during CRH test of 47 patients, including controls (*n* = 18), and patients (P1 + P2, *n* = 29), subdivided into subjects who passed CRH test (defined as cortisol concentrations above 450 nmol/L (16.25 µg/dL))—P1 (*n* = 20), and those who failed CRH test—P2 (*n* = 9). Significant increase in cortisol in comparison to time 0 min (*p* < 0.05) is marked by *

Comparisons of copeptin, ACTH and cortisol secretion in comparison to initial levels (mean levels at time −15 and 0 min) are presented in Fig. 1a–c. There was a significant increase in serum copeptin in controls, at 15, 30 and 60 min after CRH, that was maximal at 30 min **(**from 3.84 ± 2.86 to 12.65 ± 24.87 pmol/L at 30 min, *p* < 0.05). In contrast, there was no significant change in copeptin concentrations in P1 and P2 (Fig. 1a). In controls and group P1, there was a significant and virtually identical increase in ACTH and cortisol concentrations (Fig. 1b, c). As expected, ACTH and cortisol levels were lower in group P2, i.e., in subjects, who failed CRH test.

**Fig. 2:**
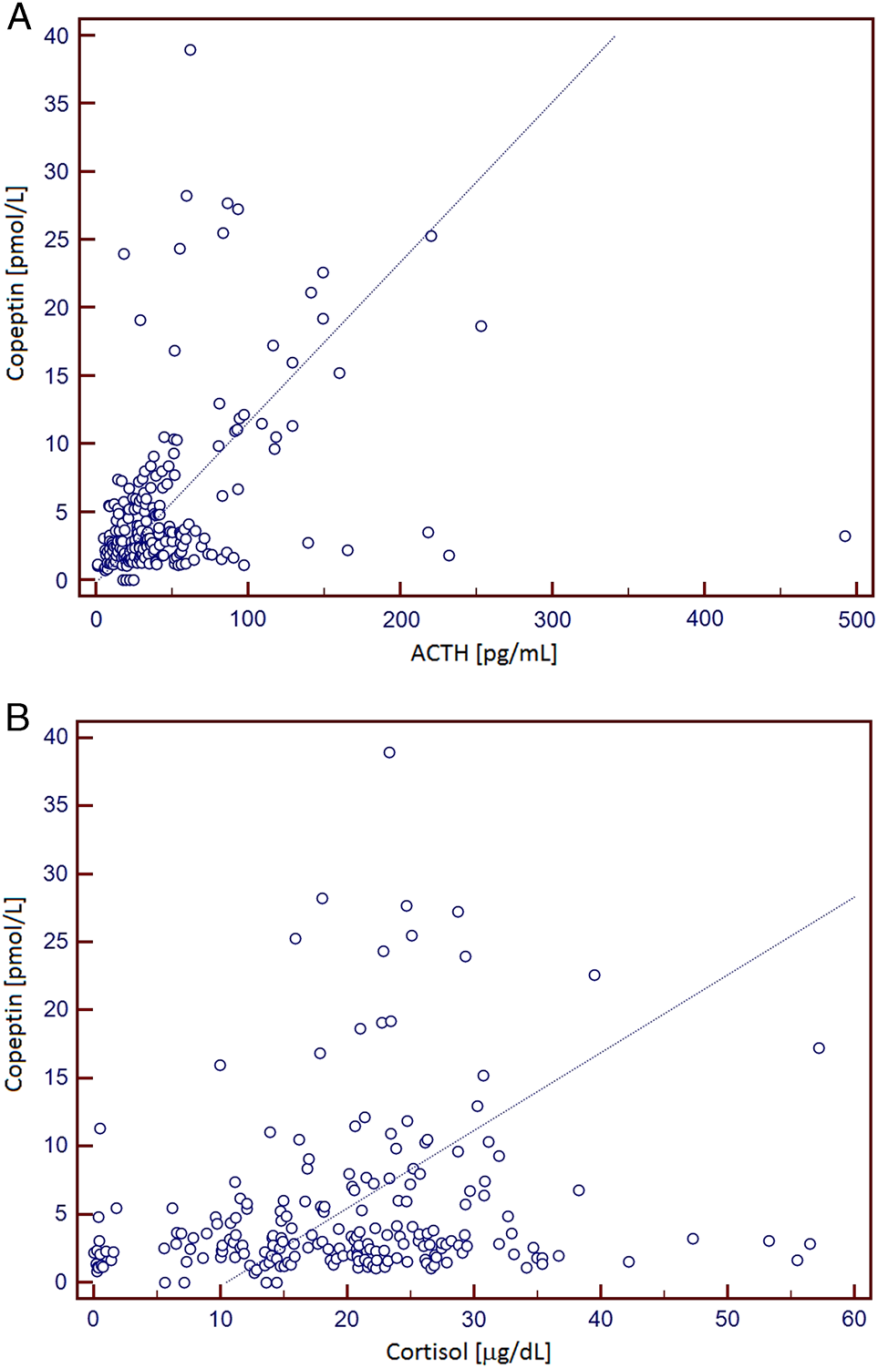
**a** Spearman rank correlation between copeptin and ACTH (all time points combined) during CRH test (*p* < 0.001, *r* = 0.41). **b** Spearman rank correlation between copeptin and cortisol (all time points combined) during CRH test (*p* = 0.0038, *r* = 0.201)

Correlation analysis between copeptin and ACTH and cortisol (all time points combined—Fig. 2a, b) revealed a significant (*p* < 0.001), though moderate (*r* = 0.41) correlation between copeptin and ACTH concentrations, and still significant (*p* = 0.0038), but weak (*r* = 0.201) correlation between copeptin and cortisol.

## Discussion

There are previous data on synergistic action of both CRH and AVP on ACTH release. Inder et al. [23] demonstrated a respectively highly significant rise in cortisol, ACTH, AVP and CRH following hypoglycaemia. They interestingly demonstrated as well increases in AVP following administration of ovine CRH in some subjects. In vitro studies confirm a close interaction between CRH and AVP. This is exemplified by treatment of pituitary cells for 1 h with CRH, which increases the percentage of corticotrophs that bind AVP [24]. The reverse phenomenon also occurs; treatment of pituitary cells for 1 h with AVP increased CRH binding per corticotroph and the percentage of cells that bound CRH [25]. More recent studies [26] demonstrated that vasopressin V1b receptor and CRH receptor 1 are capable of forming constitutive homo- and heterodimers, and that this interaction does not affect the binding properties of the receptors. Clinical studies suggest that AVP rather enhances the effects of CRH, while isolated stimulatory effects of AVP on ACTH release are much more modest, i.e., relatively high intravenous AVP doses are necessary to stimulate ACTH and cortisol secretion in healthy volunteers [27]. On the other hand, ACTH responses to ITT are higher than maximal ACTH responses to CRH [28]. Comparable to intramuscular glucagon injection, ACTH responses were higher, than after isolated administration of either human CRH or AVP [29]. Differential effects of CRH and AVP on corticotrophs, were confirmed by recent electrophysiological studies, that demonstrated that corticotroph cells of the anterior pituitary are electrically excitable. In corticotrophs this bursting is primarily controlled by activation of the CRH-signalling pathways, whereas AVP promotes an increase in action potential frequency [30, 31].

Yet, while “classical” dynamic studies of pituitary function (i.e., ITT or GST) result in simultaneous release of both CRH and AVP, in our study we investigated isolated effects of CRH on AVP/copeptin secretion. Our study demonstrates for the first time that CRH stimulates copeptin release; thus, this phenomenon is likely to be, at least partially, responsible for an increase in copeptin concentrations observed during ITT or GST that was noted before [17, 20]. Simultaneously we also observed a significant, though rather moderate (*r* = 0.406, *p* < 0.001), correlation between copeptin and plasma ACTH concentrations. The observed increase in serum copeptin in the control group was simultaneous with ACTH, similar to observations of Demiralay et al. [32], who also observed simultaneous release of copeptin and ACTH during stress, i.e., during CCK-4-induced panic symptoms. Yet, CRH-dependent stimulation of ACTH is largely independent of AVP/copeptin, as in a group of subjects with a history of pituitary disease, but normal ACTH–cortisol responses to CRH, we demonstrated no significant increase in copeptin despite highly significant ACTH–cortisol response of the same magnitude as in healthy controls (i.e., approximately a fourfold increase in a mean ACTH concentrations). On the other hand in a group of subjects, that failed to obtain satisfactory ACTH/cortisol release during CRH, we had a significant number of subjects (five out of nine) with panhypopituitarism and diabetes insipidus (DI). In our opinion, the presence of diabetes insipidus, that is associated with low AVP/copeptin secretion, was the main factor responsible for lower copeptin concentrations in that group. We therefore confirm that subjects with a history of pituitary disease have lower copeptin secretion after CRH stimulation, even in the setting of the absence of clinically significant abnormalities in ACTH–cortisol axis. Lower copeptin secretion was also seen in subjects with mildly impaired pituitary function during ITT [18], as well as during GST [20]. Hence, we can conclude that copeptin appears to be a sensitive marker of alterations of anterior pituitary function, even below a threshold that warrants glucocorticoid substitution. The reason for this phenomenon remains to be fully elucidated. There are, however, data that AVP secretion in the response to hypoglycaemia is blunted by somatostatin-induced inhibition of growth hormone secretion [33, 34]. There is a possibility that some subjects in group P1 had a subtle growth hormone deficiency. Hence, we can speculate whether GH deficiency might contribute to blunted AVP/copeptin response after CRH stimulation. This hypothesis, however, requires further study (e.g., assessment of copeptin secretion after CRH in healthy subjects before and after somastostatin). Our subjects, however, were not formally tested for GH deficiency, as GH treatment in adults is not covered by the Polish state insurance.

In summary, we have demonstrated that CRH is able to stimulate copeptin release in healthy controls suggesting a direct interaction of CRH and AVP/vasopressin. Interestingly, this relation is altered already in the group of pituitary patients who pass the standard CRH test in terms of satisfactory ACTH and cortisol secretion. In our opinion, this indicates that the CRH–ACTH–cortisol response is largely independent from the AVP system, yet simultaneously, CRH–AVP interaction reflected by copeptin may be much more sensitive to reveal subtle alterations in the regulation of pituitary function.
